# TROPION-Lung10: a phase 3 study of datopotamab deruxtecan and rilvegostomig in patients with treatment-naïve locally advanced or metastatic nonsquamous non-small cell lung cancer with high PD-L1 expression and without actionable genomic alterations

**DOI:** 10.3389/fonc.2025.1721624

**Published:** 2026-01-26

**Authors:** Thomas Newsom-Davis, Barbara Melosky, Rebecca S. Heist, Shun Lu, Giulia Pasello, Xiwei Chen, Marta Stachowiak, Pavlo Lyfar, Alessandra Forcina, Suresh S. Ramalingam

**Affiliations:** 1 Oncology Department, Chelsea and Westminster Hospital, London, United Kingdom; 2 Division of Medical Oncology, BC Cancer-Vancouver Centre, Vancouver, BC, Canada; 3 Department of Medicine, Massachusetts General Hospital, Boston, MA, United States; 4 Department of Medical Oncology, Shanghai Lung Cancer Center, Shanghai, China; 5 Medical Oncology 2, Veneto Institute of Oncology, Padova, Italy; 6 Biostatistics, AstraZeneca, Wilmington, DE, United States; 7 Clinical Development, AstraZeneca, Warsaw, Poland; 8 Global Development, AstraZeneca, Mississauga, ON, Canada; 9 Oncology R&D, AstraZeneca, Cambridge, United Kingdom; 10 Department of Hematology and Medical Oncology, Winship Cancer Institute of Emory University Atlanta, Atlanta, GA, United States

**Keywords:** antibody–drug conjugate, datopotamab deruxtecan, non-small cell lung cancer, programmed cell death ligand-1, rilvegostomig, TIGIT, topoisomerase I, TROPION-Lung10

## Abstract

**Background:**

Immunotherapy targeting the programmed cell death (ligand)-1 (PD-[L]1) pathway has improved outcomes in patients with advanced/metastatic non-small cell lung cancer (NSCLC) without actionable genomic alterations (AGAs), especially those with high PD-L1 expression (≥50% of tumor cells [TC]). However, some patients have primary or acquired resistance to treatment and new therapeutic strategies are needed to address this. Datopotamab deruxtecan (Dato-DXd), a trophoblast cell surface antigen 2 (TROP2)-directed antibody–drug conjugate, and rilvegostomig, a bispecific anti-PD-1/anti-TIGIT antibody, have shown promising efficacy and manageable safety profiles in patients with advanced or metastatic NSCLC.

**Methods and design:**

TROPION-Lung10 (NCT06357533) is a phase 3, open-label, multicenter, randomized study evaluating the efficacy and safety of first-line Dato-DXd plus rilvegostomig versus standard-of-care pembrolizumab in patients with advanced/metastatic nonsquamous NSCLC with PD-L1 TC expression ≥50% and without AGAs. Approximately 675 adults with nonsquamous stage IIIB/C or IV NSCLC not amenable to curative surgery or definitive chemoradiation, PD-L1 TC ≥50%, and no AGAs will be enrolled. Patients will be randomized (2:1:2) to receive Dato-DXd (6 mg/kg intravenously [IV] every 3 weeks [Q3W]) plus rilvegostomig (750 mg IV Q3W), rilvegostomig alone (750 mg IV Q3W), or pembrolizumab (200 mg IV Q3W for up to 35 cycles/24 months). The dual primary endpoints are progression-free survival (PFS) by blinded independent central review (BICR) per Response Evaluation Criteria in Solid Tumors version 1.1 (RECIST v1.1) and overall survival (OS) in the TROP2 normalized membrane ratio (NMR) biomarker-positive population for Dato-DXd plus rilvegostomig versus pembrolizumab. The key secondary endpoints are PFS by BICR per RECIST v1.1 and OS in the full analysis set (FAS). Other secondary endpoints include the objective response rate and duration of response by BICR per RECIST v1.1, PFS2, patient-reported outcomes in the TROP2 NMR-positive population and FAS, and safety.

**Discussion:**

TROPION-Lung10 will assess first-line Dato-DXd plus rilvegostomig in patients with advanced/metastatic NSCLC with high PD-L1 expression and without AGAs.

**Clinical trial registration:**

## Plain language summary

Most patients diagnosed with non-small cell lung cancer (NSCLC) have disease that has spread from its original site to other parts of the body (advanced/metastatic disease). For patients whose tumors express a protein called programmed cell death ligand-1 (PD-L1) on ≥50% of their tumor cells (TC ≥ 50%) and who do not have genetic changes for which there are approved targeted therapies (actionable genomic alterations), the preferred first-line treatment option is immunotherapy, which targets the immune system to help the body fight cancer. However, some patients do not respond to treatment, or it stops working, and new treatment options are needed. 

Datopotamab deruxtecan (Dato-DXd) and rilvegostomig are drugs that have shown promising antitumor activity in patients with advanced/metastatic NSCLC. Dato-DXd is an antibody–drug conjugate consisting of an antibody (datopotamab) and an anticancer drug (DXd), joined via a plasma-stable cleavable linker. Dato-DXd is directed toward a protein called “TROP2” on the surface of tumor cells. There is evidence that measuring the amount of TROP2 on the tumor cell surface relative to the inside of the cell (called TROP2 normalized membrane ratio or TROP2 NMR) might be able to identify patients who are more likely to benefit from treatment with Dato-DXd. Rilvegostomig is an antibody that blocks two proteins, PD-1 and TIGIT, to help the immune system kill cancer cells.

The TROPION-Lung10 study will assess if treatment with Dato-DXd plus rilvegostomig can improve outcomes for patients with advanced/metastatic NSCLC compared with pembrolizumab, which is the current standard treatment. Patients included in the study will have a type of advanced/metastatic NSCLC known as “non-squamous” NSCLC, with PD-L1 TC ≥50% and without actionable genomic alterations. Patients must not have received any previous treatment for advanced/metastatic NSCLC. Approximately 675 patients will be randomly assigned to receive either Dato-DXd plus rilvegostomig, rilvegostomig alone, or pembrolizumab. The main aim of the study is to see how long patients in the TROP2 NMR-positive group live (overall survival), and how long they live without their cancer growing or spreading (progression-free survival) with Dato-DXd plus rilvegostomig treatment compared with patients who receive pembrolizumab.

## 1 Introduction

Non-small cell lung cancer (NSCLC) accounts for approximately 85% of all lung cancers (1), with most patients having distant metastases at the time of initial diagnosis (2). Immune checkpoint inhibitors have become an important treatment option for patients with advanced/metastatic NSCLC without actionable genomic alterations (AGAs) (3, 4). First-line treatment with programmed cell death 1/ligand-1 (PD-1/PD-L1) inhibitors has improved outcomes for these patients, especially for those with high PD-L1 expression (5–14). Multiple treatment options are available for patients with advanced NSCLC and PD-L1 expression on at least 50% of tumor cells (TC ≥50%), including anti-PD-1/PD-L1 monotherapy (3, 4). However, more than half of the patients do not respond to first-line anti-PD-1/PD-L1 monotherapy (9–13), and the 5-year overall survival (OS) rates range from 22% to 32% (12–14). Therefore, new therapeutic strategies that may improve the efficacy of first-line treatment for patients with advanced/metastatic NSCLC with PD-L1 TC ≥50% and approaches to identify patients who may respond are needed.

Datopotamab deruxtecan (Dato-DXd) and rilvegostomig are anticancer agents that have shown promising efficacy in patients with advanced/metastatic NSCLC. Dato-DXd is an antibody–drug conjugate composed of a humanized anti-trophoblast cell surface antigen 2 (TROP2) immunoglobulin G1 monoclonal antibody conjugated to a highly potent topoisomerase I inhibitor payload via a plasma-stable, tetrapeptide-based, tumor-selective and cleavable linker (15). Dato-DXd is approved for the treatment of patients with locally advanced/metastatic *EGFR*-mutated NSCLC who have received prior *EGFR*-directed therapy and platinum-based chemotherapy (16). Approval was based on data from the phase 2 TROPION-Lung05 study (NCT04484142) and supported by data from the phase 3 TROPION-Lung01 study (NCT04656652) (17, 18). In a pooled analysis of patients from TROPION-Lung05 and TROPION-Lung01 with previously treated *EGFR*-mutated NSCLC, Dato-DXd demonstrated a confirmed objective response rate (ORR) of 43% (95% confidence interval [CI]: 34–52) and a median duration of response (DoR) of 7.0 months (95% CI: 4.2–9.8) (19). Moreover, in TROPION-Lung01, Dato-DXd monotherapy significantly improved progression-free survival (PFS) compared with docetaxel (median PFS: 4.4 vs. 3.7 months; hazard ratio [HR] 0.75 [95% CI: 0.62–0.91]) in patients with pretreated advanced/metastatic NSCLC with or without AGAs, which was driven by the benefit in patients with nonsquamous histology (18). Notably, an exploratory analysis of TROPION-Lung01 showed that the TROP2 normalized membrane ratio (NMR), assessed by a computational pathology-based approach called quantitative continuous scoring, showed potential as a predictive biomarker for response to Dato-DXd (20). Patients receiving Dato-DXd who had TROP2 NMR-positive (NMR+) tumors had higher ORR and longer PFS than those with TROP2 NMR-negative (NMR−) tumors, including a focused subgroup of patients with nonsquamous histology without AGAs (20).

Dato-DXd in combination with anti-PD-1/PD-L1 therapy, with or without chemotherapy, has also shown activity as a first-line treatment for advanced/metastatic NSCLC. In the phase 1b TROPION-Lung02 study (NCT04526691) in patients with nonsquamous advanced NSCLC, first-line Dato-DXd plus pembrolizumab with or without chemotherapy demonstrated ORRs of 52% and 57% and disease control rates (DCRs) of 88% and 91%, respectively; the median duration of response was 24.9 and 18.0 months, respectively (21). An exploratory retrospective analysis of TROPION-Lung02 showed that patients with TROP2 NMR+ tumors had prolonged PFS and OS compared to those with NMR− tumors, further demonstrating its potential as a predictive biomarker in this setting (21). In the ongoing phase 1b TROPION-Lung04 study (NCT04612751), first-line Dato-DXd in combination with durvalumab, with or without carboplatin, demonstrated ORRs of 50% and 77% and DCRs of 93% and 92%, respectively (22). In both studies, no new safety signals were reported, and the safety profiles of the combination therapies were as expected based on the profile of each individual agent (21, 22).

Rilvegostomig is a monovalent, Fc-reduced, bispecific, humanized monoclonal immunoglobulin G1 antibody that targets both PD-1 and T cell immunoglobulin and immunoreceptor tyrosine-based inhibitory motif domain (TIGIT) receptors (23). TIGIT is a coinhibitory receptor expressed on activated T and natural killer cells (24). The bispecific mechanism of action of rilvegostomig enables the coordinated and synchronous inhibition of both PD-1 and TIGIT and, as shown in preclinical studies, has the potential to enhance antitumor immune responses compared with anti-PD-1 or anti-TIGIT monotherapies or combinations of these individual monoclonal antibodies (24–28). In the phase 1/2 ARTEMIDE-01 study (NCT04995523), rilvegostomig showed preliminary efficacy, with an ORR of 62% and an acceptable safety profile in patients with advanced/metastatic NSCLC with PD-L1 TC ≥50% who were naïve to immune checkpoint inhibitors (23). Notably, first-line treatment with a combination of Dato-DXd and rilvegostomig demonstrated promising efficacy and safety in cohort 5 of the TROPION-Lung04 study; in patients with nonsquamous histology, the ORR was 62%, and the DCR was 100% (median DoR was not reached) (29). The safety profile of this combination was consistent with the known safety profiles of each individual agent, and no new safety signals were reported (29). Although the rate of adjudicated drug-related interstitial lung disease (ILD; 12.5%) (29) was slightly higher than that reported in studies of Dato-DXd monotherapy in advanced/metastatic NSCLC (4%–9%) (17, 18, 30), none of these events were grade 4 or 5.

Together, these data suggest that combining the directed cytotoxicity of Dato-DXd with immune checkpoint inhibition of rilvegostomig has the potential to improve outcomes for patients with advanced/metastatic NSCLC. TROPION-Lung10 (NCT06357533) is evaluating the efficacy and safety of Dato-DXd plus rilvegostomig as a first-line treatment for patients with advanced/metastatic nonsquamous NSCLC with high PD-L1 expression (TC ≥50%) and no AGAs versus standard-of-care pembrolizumab monotherapy.

## 2 Methods and analysis

### 2.1 Study design

**Figure 1:**
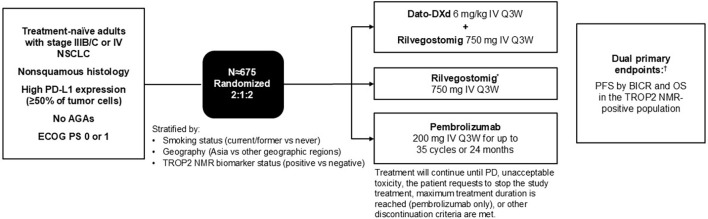
TROPION-Lung10 (NCT06357533) study design. *The rilvegostomig monotherapy arm will be used to assess the contribution of components of the experimental arm. ^†^The dual primary endpoints will be assessed for Dato-DXd + rilvegostomig versus pembrolizumab monotherapy. AGA, actionable genomic alteration; BICR, blinded independent central review; Dato-DXd, datopotamab deruxtecan; ECOG PS, Eastern Cooperative Oncology Group performance status; IV, intravenously; NMR, normalized membrane ratio; NSCLC, non-small cell lung cancer; OS, overall survival; PD, disease progression; PD-L1, programmed cell death ligand-1; PFS, progression-free survival; Q3W, every 3 weeks; TROP2, trophoblast cell surface antigen-2.

TROPION-Lung10 is a phase 3, randomized, open-label, multicenter study (**Figure 1**). Approximately 675 patients will be randomized in a 2:1:2 ratio to receive Dato-DXd (6 mg/kg intravenously [IV] every 3 weeks [Q3W]) plus rilvegostomig (750 mg IV Q3W), rilvegostomig monotherapy (750 mg IV Q3W), or pembrolizumab monotherapy (200 mg IV Q3W). The rilvegostomig monotherapy arm is included to assess the contribution of the components of the experimental arm (Dato-DXd plus rilvegostomig). Randomization will be stratified by smoking status (current/former vs. never), geography (Asia vs. other geographic regions), and TROP2 NMR status (TROP2 NMR+ vs. TROP2 NMR−). Patients will receive treatment until disease progression, unacceptable toxicity, patient requests to stop the study treatment, or other discontinuation criteria are met. Patients receiving pembrolizumab may receive treatment for a maximum of 35 cycles/24 months. No crossover between the study arms will be allowed.

### 2.2 Eligibility criteria

The key inclusion and exclusion criteria for TROPION-Lung10 are summarized in **Table 1**. Eligible patients are aged ≥18 years and have histologically or cytologically documented nonsquamous stage IIIB/C or IV NSCLC (based on the American Joint Committee on Cancer Staging Manual, 8th Edition) that is not amenable to curative surgery or definitive chemoradiation. Patients must have an Eastern Cooperative Oncology Group (ECOG) performance status of 0 or 1, measurable disease per Response Evaluation Criteria in Solid Tumors version 1.1 (RECIST v1.1), no sensitizing *EGFR* mutations or *ALK* and *ROS1* rearrangements, and no documented tumor genomic alterations in any other actionable driver oncogenes for which there are locally approved and available targeted first-line therapies. Provision of a tumor sample to determine PD-L1 status, TROP2 NMR status, and other biomarkers prior to randomization is mandatory. Patients must have tumors with PD-L1 expression in ≥50% of TCs. TROP2 NMR status will be determined using a TROP2 NMR assay. Prior chemotherapy or systemic therapy for stage IIIB/C or IV NSCLC is not permitted. Patients with a history of another primary malignancy within 3 years of enrolment or any primary immunodeficiency will be excluded. Other exclusion criteria include: severe or uncontrolled systemic diseases; clinically significant third-space fluid retention not amenable to repeated drainage; history of, current, or suspected ILD/pneumonitis; significantly compromised pulmonary function; spinal cord compression; brain metastases (unless treated, no longer symptomatic, and radiologically stable); history of leptomeningeal carcinomatosis; clinically significant corneal disease; active infection, including tuberculosis or hepatitis A, B, or C virus; or uncontrolled infection with human immunodeficiency virus.

**Table 1:** Key inclusion and exclusion criteria.

Key inclusion criteria
• Histologically or cytologically documented nonsquamous NSCLC • Stage IIIB/C or stage IV NSCLC (based on the AJCC Staging Manual, 8th Edition) not amenable to curative surgery or definitive chemoradiation • No prior chemotherapy or other systemic therapy for stage IIIB/C or IV NSCLC • Absence of sensitizing *EGFR* mutations and *ALK* and *ROS1* rearrangements, and absence of documented tumor genomic alterations in any other actionable driver oncogenes for which there are locally approved and available targeted first-line therapies • Eastern Cooperative Oncology Group performance status of 0 or 1 • Minimum life expectancy of 12 weeks • Provision of a tumor sample prior to the start of screening to determine PD-L1 status, TROP2 NMR status, and other biomarkers prior to randomization • PD-L1 expression on ≥50% of tumor cells • Measurable disease per RECIST v1.1 • Adequate bone marrow reserve and organ function within 7 days before the first dose of study intervention

Key exclusion criteria
• Severe or uncontrolled systemic diseases, history of allogenic organ transplant, or active or prior documented autoimmune or inflammatory disorders • History of another primary malignancy within 3 years • Persistent toxicities caused by previous anticancer therapy • Spinal cord compression, or brain metastases unless treated, no longer symptomatic, radiologically stable, and that does not require treatment with corticosteroids or anticonvulsants • History of leptomeningeal carcinomatosis • Clinically significant third-space fluid retention not amenable for repeated drainage • Clinically significant corneal disease • Active infection including with tuberculosis, HBV, HCV, or HAV, or uncontrolled HIV infection • History of active primary immunodeficiency • History of non-infectious ILD/pneumonitis including radiation pneumonitis that required steroids, or current or suspected ILD/pneumonitis • Significantly compromised pulmonary function • Resting electrocardiogram with clinically abnormal findings • Prior exposure to: o Any anti-TIGIT or anti-PD-1/-PD-L1 therapy or an antibody targeting any other immuno-regulatory receptors or mechanisms o Therapeutic anticancer vaccines o Any regimen including ADCs containing a chemotherapeutic agent targeting topoisomerase I o TROP2-targeted therapy • Any concurrent anticancer treatment

ADC, antibody-drug conjugate; AJCC, American Joint Committee on Cancer; Dato-DXd, datopotamab deruxtecan; EGFR, epidermal growth factor receptor; HAV/HBV/HCV, hepatitis A/B/C virus; HIV, human immunodeficiency virus; ILD, interstitial lung disease; NMR, normalized membrane ratio; NSCLC, non-small cell lung cancer; PD-(L)1, programmed cell death (ligand) 1; RECIST v1.1; Response Evaluation Criteria in Solid Tumors version 1,1; TIGIT, T cell immunoglobulin and immunoreceptor tyrosine-based inhibitory motif domain; TROP2, trophoblast cell surface antigen-2.

### 2.3 Objectives and endpoints

The study endpoints are summarized in **Table 2**. The dual primary endpoints are PFS by blinded independent central review (BICR) per RECIST v1.1 and OS in the TROP2 NMR+ population compared between Dato-DXd plus rilvegostomig and pembrolizumab monotherapy. PFS is defined as the time from randomization until disease progression, as assessed by BICR per RECIST v1.1, or death due to any cause. OS is defined as the time from randomization to death due to any cause.

**Table 2:** TROPION-Lung10 endpoints.

Endpoints	Dato-DXd + rilvegostomig vs pembrolizumab		
Dual primary endpoints	• PFS per RECIST v1.1 by BICR in the TROP2 NMR+ population • OS in the TROP2 NMR+ population		

	Dato-DXd + rilvegostomig vs pembrolizumab	Rilvegostomig monotherapy vs pembrolizumab	Dato-DXd + rilvegostomig vs rilvegostomig monotherapy
Secondary endpoints	• PFS by BICR per RECIST v1.1 in the FAS • OS in the FAS • ORR and DoR by BICR per RECIST v1.1 in the TROP2 NMR+ population and FAS • PFS2 in the TROP2 NMR+ population and FAS • Patient-reported outcomes in the TROP2 NMR+ population and FAS	• PFS by BICR per RECIST v1.1 in the TROP2 NMR+ population and FAS • OS in the TROP2 NMR+ population and FAS • ORR and DoR by BICR per RECIST v1.1 in the TROP2 NMR+ population and FAS	
	• Safety and tolerability	–	
	• Pharmacokinetics of Dato-DXd + rilvegostomig and rilvegostomig monotherapy • Immunogenicity of Dato-DXd + rilvegostomig and rilvegostomig monotherapy		

TROP2 NMR status will be assessed using the TROP2 NMR assay.

BICR, blinded independent central review; Dato-DXd, datopotamab deruxtecan; DoR, duration of response; FAS, full analysis set; NMR, normalized membrane ratio; ORR, objective response rate; OS, overall survival; PFS, progression-free survival; PFS2, time to second progression or death; RECIST v1.1, Response Evaluation Criteria in Solid Tumors version 1.1; TROP2, trophoblast cell surface antigen 2.

The key secondary endpoints are PFS by BICR per RECIST v1.1 and OS in the full analysis set (FAS) compared between Dato-DXd plus rilvegostomig and pembrolizumab monotherapy. A summary of the endpoints pertaining to each treatment group comparison is presented in **Table 2**. Other secondary endpoints for Dato-DXd plus rilvegostomig versus pembrolizumab in the TROP2 NMR+ population and the FAS include ORR and DoR by BICR per RECIST v1.1, time to second progression or death (PFS2), defined as the time from randomization to the earliest of a progression event (following initial investigator-assessed progression) after first subsequent therapy or death, and patient-reported outcomes (PROs; time to deterioration in pulmonary symptoms and overall lung cancer symptoms, physical functioning, and global health status/quality of life [GHS/QoL]). PFS, OS, ORR, and DoR will also be analyzed in the TROP2 NMR+ population and the FAS for rilvegostomig monotherapy versus pembrolizumab and Dato-DXd plus rilvegostomig versus rilvegostomig monotherapy. Finally, the safety and tolerability parameters (examined for Dato-DXd plus rilvegostomig and rilvegostomig monotherapy versus pembrolizumab monotherapy) will include adverse events (AEs) graded according to the Common Terminology Criteria for Adverse Events version 5.0. The pharmacokinetics and immunogenicity of Dato-DXd plus rilvegostomig and rilvegostomig monotherapy will also be assessed.

### 2.4 Study procedures and assessments

Randomization will occur within 28 days of signing the main informed consent form. Tumor assessments by computed tomography (CT; preferred) or magnetic resonance imaging (MRI) will be performed at baseline (within 28 days before randomization) and at regular intervals during the study treatment. A brain scan by MRI (preferred) or CT must also be performed at baseline, as close as possible to the start of treatment, and for patients with brain metastases, at every tumor assessment scan.

Patients will be assessed for PFS2 at regular intervals after initial objective disease progression until the second progression on subsequent treatment, death, withdrawal of consent, or the end of the study. OS assessments will be conducted following objective disease progression or treatment discontinuation (whichever occurs first) until death, withdrawal of consent, or the end of the study period. Secondary PRO endpoints will be assessed via electronic questionnaires administered on Day 1 of Cycle 1, prior to the first dose of study treatment, and throughout the study period until 18 weeks after disease progression. The time to deterioration in pulmonary symptoms and overall lung cancer symptoms, physical functioning, and GHS/QoL will be measured using the NSCLC symptom assessment questionnaire, the Patient-Reported Outcomes Measurement Information System Physical Function Short Form 8c, and the European Organisation for Research and Treatment of Cancer questionnaire Item Library 172, respectively. Physical examinations and laboratory assessments will be performed, ECOG performance status and vital signs assessed, and electrocardiograms recorded on Day 1 of Cycle 1, throughout treatment, at the end of treatment, and during the safety follow-up period.

AEs will be collected throughout the treatment period and during the safety follow-up period, and ILD/pneumonitis events will be followed up beyond this period until resolution. AEs related to Dato-DXd and rilvegostomig will be managed according to the toxicity management guidelines for each drug. Dose delays are permitted for all the study interventions. Dose reductions are permitted for Dato-DXd but not for rilvegostomig or pembrolizumab. In cases of suspected ILD/pneumonitis, the study treatment should be delayed while a full investigation is performed. All potential cases of ILD/pneumonitis will be reviewed by an independent ILD Adjudication Committee. Ophthalmological assessments will be performed as clinically indicated throughout the treatment. Patients receiving Dato-DXd are advised to use artificial tears daily and avoid contact lenses. In a daily oral care plan provided to all patients receiving Dato-DXd, the daily use of prophylaxis with a steroid-containing mouthwash is highly recommended, and prophylactic cryotherapy (ice chips or ice water held in the mouth throughout the infusion) is also advised. Prophylactic anti-emetic agents prior to Dato-DXd infusion and on subsequent days as needed, are highly recommended.

Blood samples will be collected at various time points throughout the treatment and will be used to analyze the pharmacokinetics and immunogenicity of Dato-DXd and rilvegostomig in combination and rilvegostomig monotherapy.

### 2.5 Statistical methods

Approximately 675 patients will be randomized. The FAS will include all randomized patients. The TROP2 NMR+ population will include all patients with TROP2 NMR+ status who are randomized in the study. The FAS and TROP2 NMR+ populations will be used for efficacy analyses and secondary PRO endpoints. The safety analysis set will include all patients who receive at least one dose of the study intervention and for whom any post-dose data are available. Pharmacokinetics will be assessed in all patients who receive at least one dose of Dato-DXd or rilvegostomig and for whom there is at least one reportable pharmacokinetic concentration. Immunogenicity will be assessed in patients in the safety analysis set with at least one non-missing Dato-DXd or rilvegostomig anti-drug antibody result at any time.

The study is powered to demonstrate the superiority of Dato-DXd plus rilvegostomig versus pembrolizumab monotherapy, as measured by the dual primary endpoints of PFS by BICR and OS in the TROP2 NMR+ population. A multiple testing procedure for the dual primary and key secondary endpoints will be implemented. The study will be considered positive if either the PFS by BICR or the OS analysis in the TROP2 NMR+ population favors Dato-DXd plus rilvegostomig versus pembrolizumab.

Time-to-event endpoints will be compared between arms using a log-rank test stratified by smoking status, TROP2 NMR status, and geographic location and presented using Kaplan–Meier estimates. The HRs and associated 95% CIs and p-values will be estimated using a stratified Cox proportional hazards model. Subgroup analyses of PFS and OS in the TROP2 NMR+ population and FAS will be conducted. ORR will be analyzed using a stratified Cochran–Mantel–Haenszel test, with 95% CIs estimated using the Miettinen–Nurminen method. The DoR will be summarized and presented using Kaplan–Meier estimates in responding patients. Safety and tolerability will be summarized descriptively.

## 3 Discussion

Although first-line immunotherapy targeting the PD-1/PD-L1 pathway has improved outcomes for patients with advanced/metastatic NSCLC without AGAs, novel treatment strategies are needed to overcome primary resistance to treatment and to identify patients who may respond.

TROPION-Lung10 will investigate the efficacy and safety of the combination of two anticancer agents, Dato-DXd and rilvegostomig, versus standard-of-care pembrolizumab in patients with advanced/metastatic nonsquamous NSCLC with high PD-L1 expression (TC ≥50%) and without AGAs. The directed cytotoxicity of Dato-DXd combined with the immune checkpoint inhibition of rilvegostomig has the potential to improve outcomes in this setting. The results of the TROPION-Lung01, TROPION-Lung02, and TROPION-Lung04 studies demonstrated the promising efficacy and manageable safety of Dato-DXd alone or in combination with immunotherapy, with or without chemotherapy (18, 21, 22). In addition to these studies, favorable preliminary evidence of the efficacy and safety of rilvegostomig has been observed in the ARTEMIDE-01 study (23), and notably, the promising efficacy and safety of the combination of Dato-DXd and rilvegostomig has been demonstrated in cohort 5 of the TROPION-Lung04 study (29). Additionally, the use of the TROP2 NMR biomarker may enable the identification of patients more likely to achieve better responses to treatment and will build on early evidence of its utility in predicting responses to Dato-DXd in patients with advanced/metastatic NSCLC (20, 21). These lines of evidence provide a rationale for the TROPION-Lung10 study.

Additionally, adjuvant treatment with Dato-DXd plus rilvegostomig is being investigated in patients with early stage adenocarcinoma NSCLC in the ongoing phase 3 TROPION-Lung12 study (NCT06564844). Several other studies investigating first-line Dato-DXd in combination with other anticancer agents in advanced/metastatic NSCLC are ongoing, including the phase 3 TROPION-Lung07 (NCT05555732), TROPION-Lung08 (NCT05215340), and AVANZAR (NCT05687266) trials. Enrolment in TROPION-Lung10 began in April 2024 and is ongoing.

## References

[1] Duma N Santana-Davila R Molina JR . Non-small cell lung cancer: epidemiology, screening, diagnosis, and treatment. Mayo Clin Proc. (2019) 94:1623–40. doi: 10.1016/j.mayocp.2019.01.013, PMID: 31378236

[2] Simeone JC Nordstrom BL Patel K Klein AB . Treatment patterns and overall survival in metastatic non-small-cell lung cancer in a real-world, US setting. Future Oncol. (2019) 15:3491–502. doi: 10.2217/fon-2019-0348, PMID: 31497994

[3] Hendriks LE Kerr KM Menis J Mok TS Nestle U Passaro A . Non-oncogene-addicted metastatic non-small-cell lung cancer: ESMO Clinical Practice Guideline for diagnosis, treatment and follow-up. Ann Oncol. (2023) 34:358–76. doi: 10.1016/j.annonc.2022.12.013, PMID: 36669645

[4] Leighl NB Ismaila N Durm G Florez N Freeman-Daily J Pellini B . Therapy for stage IV non-small cell lung cancer without driver alterations: ASCO living guideline, version 2024.3. J Clin Oncol. (2025) 43:e17–30. doi: 10.1200/JCO-24-02786, PMID: 40014838

[5] Gandhi L Rodriguez-Abreu D Gadgeel S Esteban E Felip E De Angelis F . Pembrolizumab plus chemotherapy in metastatic non-small-cell lung cancer. N Engl J Med. (2018) 378:2078–92. doi: 10.1056/NEJMoa1801005, PMID: 29658856

[6] Johnson ML Cho BC Luft A Alatorre-Alexander J Geater SL Laktionov K . Durvalumab with or without tremelimumab in combination with chemotherapy as first-line therapy for metastatic non-small-cell lung cancer: the phase III POSEIDON study. J Clin Oncol. (2023) 41:1213–27. doi: 10.1200/JCO.22.00975, PMID: 36327426 PMC9937097

[7] Novello S Kowalski DM Luft A Gumus M Vicente D Mazieres J . Pembrolizumab plus chemotherapy in squamous non-small-cell lung cancer: 5-year update of the phase III KEYNOTE-407 Study. J Clin Oncol. (2023) 41:1999–2006. doi: 10.1200/JCO.22.01990, PMID: 36735893 PMC10082300

[8] Garassino MC Gadgeel S Speranza G Felip E Esteban E Domine M . Pembrolizumab plus pemetrexed and platinum in nonsquamous non-small-cell lung cancer: 5-year outcomes from the phase 3 KEYNOTE-189 study. J Clin Oncol. (2023) 41:1992–8. doi: 10.1200/JCO.22.01989, PMID: 36809080 PMC10082311

[9] Sezer A Kilickap S Gumus M Bondarenko I Ozguroglu M Gogishvili M . Cemiplimab monotherapy for first-line treatment of advanced non-small-cell lung cancer with PD-L1 of at least 50%: a multicentre, open-label, global, phase 3, randomised, controlled trial. Lancet. (2021) 397:592–604. doi: 10.1016/S0140-6736(21)00228-2, PMID: 33581821

[10] Jassem J de Marinis F Giaccone G Vergnenegre A Barrios CH Morise M . Updated overall survival analysis from IMpower110: atezolizumab versus platinum-based chemotherapy in treatment-naive programmed death-ligand 1-selected NSCLC. J Thorac Oncol. (2021) 16:1872–82. doi: 10.1016/j.jtho.2021.06.019, PMID: 34265434

[11] Reck M Rodriguez-Abreu D Robinson AG Hui R Csoszi T Fulop A . Pembrolizumab versus chemotherapy for PD-L1-positive non-small-cell lung cancer. N Engl J Med. (2016) 375:1823–33. doi: 10.1056/NEJMoa1606774, PMID: 27718847

[12] Reck M Rodriguez-Abreu D Robinson AG Hui R Csoszi T Fulop A . Five-year outcomes with pembrolizumab versus chemotherapy for metastatic non-small-cell lung cancer with PD-L1 tumor proportion score ≥ 50. J Clin Oncol. (2021) 39:2339–49. doi: 10.1200/JCO.21.00174, PMID: 33872070 PMC8280089

[13] de Castro G Jr. Kudaba I Wu YL Lopes G Kowalski DM Turna HZ . Five-year outcomes with pembrolizumab versus chemotherapy as first-line therapy in patients with non-small-cell lung cancer and programmed death ligand-1 tumor proportion score ≥ 1% in the KEYNOTE-042 study. J Clin Oncol. (2023) 41:1986–91. doi: 10.1200/JCO.21.02885, PMID: 36306479 PMC10082298

[14] Kilickap S Baramidze A Sezer A Ozguroglu M Gumus M Bondarenko I . Cemiplimab monotherapy for first-line treatment of patients with advanced NSCLC with PD-L1 expression ≥50%: 5-year outcomes of EMPOWER-Lung 1. J Thorac Oncol. (2025) 20:941–54. doi: 10.1016/j.jtho.2025.03.033, PMID: 40118215

[15] Okajima D Yasuda S Maejima T Karibe T Sakurai K Aida T . Datopotamab deruxtecan, a novel TROP2-directed antibody-drug conjugate, demonstrates potent antitumor activity by efficient drug delivery to tumor cells. Mol Cancer Ther. (2021) 20:2329–40. doi: 10.1158/1535-7163.MCT-21-0206, PMID: 34413126 PMC9398094

[16] US Food and Drug Administration (FDA) . DATROWAY^®^ (datopotamab deruxtecan-dlnk) for injection, for intravenous use. Initial U.S. approval 2025. Highlights of prescribing information. Available online at: https://www.accessdata.fda.gov/drugsatfda_docs/label/2025/761464s000lbl.pdf (Accessed August 12, 2025).

[17] Sands J Ahn MJ Lisberg A Cho BC Blumenschein G Jr. Shum E . Datopotamab deruxtecan in advanced or metastatic non-small cell lung cancer with actionable genomic alterations: results from the phase II TROPION-Lung05 study. J Clin Oncol. (2025) 43:1254–65. doi: 10.1200/JCO-24-01349, PMID: 39761483 PMC11949215

[18] Ahn MJ Tanaka K Paz-Ares L Cornelissen R Girard N Pons-Tostivint E . Datopotamab deruxtecan versus docetaxel for previously treated advanced or metastatic non-small cell lung cancer: the randomized, open-label phase III TROPION-Lung01 study. J Clin Oncol. (2024) 43:260–72. doi: 10.1200/jco-24-01544, PMID: 39250535 PMC11771353

[19] Ahn MJ Lisberg A Goto Y Sands J Hong MH Paz-Ares L . A pooled analysis of datopotamab deruxtecan in patients with EGFR-mutated NSCLC. J Thorac Oncol. (2025) 29:1669–1682. doi: 10.1016/j.jtho.2025.06.002, PMID: 40516821

[20] Garassino M Sands J Paz-Ares L Lisberg A Johnson M Pérol M . Normalized membrane ratio of TROP2 by quantitative continuous scoring is predictive of clinical outcomes in TROPION-Lung01. J Thorac Oncol. (2024) 19:S2–3. doi: 10.1016/j.jtho.2024.09.015

[21] Levy BP Paz-Ares LG Lin C-C Herbert S Yang T-Y Tolcher AW . TROPION-Lung02: Datopotamab deruxtecan (Dato-DXd) plus pembrolizumab (pembro) with or without platinum chemotherapy (Pt-CT) as first-line (1L) therapy for advanced non-small cell lung cancer (aNSCLC). J Clin Oncol. (2025) 43:8501. doi: 10.1200/JCO.2025.43.16_suppl.8501

[22] Papadopoulos KP Bruno D Kitazono S Murakami S Gutierrez M Wakuda K . Datopotamab deruxtecan (Dato-DXd) + durvalumab ± carboplatin in advanced/mNSCLC: initial results from phase 1b TROPION-Lung04. J Thorac Oncol. (2023) 18:S55. doi: 10.1016/j.jtho.2023.09.043

[23] Hiltermann TJN Izumi H Cho BC Cunha S Danchaivijitr P Felip E . Efficacy and safety of rilvegostomig, an anti-PD-1/TIGIT bispecific, for CPI-naïve metastatic NSCLC with PD-L1 1-49% or ≥50%. J Thorac Oncol. (2024) 19:S33. doi: 10.1016/j.jtho.2024.09.061

[24] Chauvin JM Pagliano O Fourcade J Sun Z Wang H Sander C . TIGIT and PD-1 impair tumor antigen-specific CD8^+^ T cells in melanoma patients. J Clin Invest. (2015) 125:2046–58. doi: 10.1172/jci80445, PMID: 25866972 PMC4463210

[25] Johnston RJ Comps-Agrar L Hackney J Yu X Huseni M Yang Y . The immunoreceptor TIGIT regulates antitumor and antiviral CD8(+) T cell effector function. Cancer Cell. (2014) 26:923–37. doi: 10.1016/j.ccell.2014.10.018, PMID: 25465800

[26] Banta KL Xu X Chitre AS Au-Yeung A Takahashi C O'Gorman WE . Mechanistic convergence of the TIGIT and PD-1 inhibitory pathways necessitates co-blockade to optimize anti-tumor CD8(+) T cell responses. Immunity. (2022) 55:512–26.e9. doi: 10.1016/j.immuni.2022.02.005, PMID: 35263569 PMC9287124

[27] Lee K Malhotra D Pryts S Clancy-Thompson E Omar B Naiman B . Preclinical studies support clinical development of AZD2936, a monovalent bispecific humanized antibody targeting PD-1 and TIGIT. J Immunother Cancer. (2022) 10:A489. doi: 10.1136/jitc-2022-SITC2022.0469

[28] Chariou P Rothstein R Miller C Kar G Johnson Y Connor T . Dual blockage of PD-1 and TIGIT with rilvegostomig inhibits tumor growth in various *in vitro*/*in vivo*/ex vivo tumor models through effector CD8 activation and immune modulation. J Immunother Cancer. (2024) 12:A543. doi: 10.1136/jitc-2024-SITC2024.0483

[29] Waqar SN Cuppens K Garcia-Campelo MR Dziadziuszko R Costa EC Yang T-Y . First-line datopotamab deruxtecan (Dato-DXd) + rilvegostomig in advanced or metastatic non-small cell lung cancer: results from TROPION-Lung04 (cohort 5). J Clin Oncol. (2025) 43:8521. doi: 10.1200/JCO.2025.43.16_suppl.8521

[30] Shimizu T Sands J Yoh K Spira A Garon EB Kitazono S . First-in-human, phase I dose-escalation and dose-expansion study of trophoblast cell-surface antigen 2-directed antibody-drug conjugate datopotamab deruxtecan in non-small-cell lung cancer: TROPION-PanTumor01. J Clin Oncol. (2023) 41:4678–87. doi: 10.1200/JCO.23.00059, PMID: 37327461 PMC10564307

