# Correction: TROPION-Lung10: a phase 3 study of datopotamab deruxtecan and rilvegostomig in patients with treatment-naïve locally advanced or metastatic nonsquamous non-small cell lung cancer with high PD-L1 expression and without actionable genomic alterations

**DOI:** 10.3389/fonc.2026.1804953

**Published:** 2026-02-11

**Authors:** Frontiers Production Office

**Affiliations:** Frontiers Media SA, Lausanne, Switzerland

**Keywords:** antibody–drug conjugate, datopotamab deruxtecan, non-small cell lung cancer, programmed cell death ligand-1, rilvegostomig, TIGIT, topoisomerase I, TROPION-Lung10

The **Conflict of interest statement** was erroneously given as “TN-D has participated in advisory boards with AbbVie, Amgen, AstraZeneca, Bayer, Bristol Myers Squibb, Boehringer Ingelheim, Daiichi Sankyo, EQRx, Gilead, GlaxoSmithKline, Janssen, Eli Lilly, Merck, Merck Sharpe & Dohme, Novartis, Novocure, Pfizer, Regeneron, Roche, Sanofi, and Takeda; has been an invited speaker for Amgen, AstraZeneca, Bristol Myers Squibb, Boehringer Ingelheim, EQRx, Gilead, GlaxoSmithKline, Janssen, Eli Lilly, Novartis, Roche, and Takeda; and has been a steering committee member for AstraZeneca, Merck Sharpe & Dohme, and Roche. BM has participated in advisory boards with Amgen, AstraZeneca, Boehringer Ingelheim, Bristol Myers Squibb, GlaxoSmithKline, Janssen, Merck, Novartis, Prizer, Regeneron, Roche, and Sanofi. RH has participated in advisory boards with Amgen, AstraZeneca, Daiichi Sankyo, Biohaven, Claim, Eli Lilly, Novartis, Regeneron, Sanofi, Merck, and Gilead; and has received research funding institutional from AstraZeneca, Daiichi Sankyo, Eli Lilly, Mirati, Novartis, Corvus, and Mythic. SL has acted in an advisory role for AstraZeneca, Pfizer, Hutchison MediPharma, ZaiLab, GenomiCare, Novartis, Yuhan Corporation, Menarini, Mirati Therapeutics Inc, Daiichi Sankyo, Inc., D3 Bio Limited, Simcere, Takeda and Roche; has been an invited speaker for AstraZeneca, Roche, and Hansoh; is a member of the board of directors for Innovent Biologics, Inc.; has received research grants from AstraZeneca, Hutchison, Bristol Myers Squibb, Heng Rui, BeiGene, Roche, and Hansoh; and has participated in a Speaker’s bureau for AstraZeneca, Roche, and Hansoh. GP has participated in advisory boards for, received honoraria and speaker’s fees from, and acted as a consultant for Amgen, AstraZeneca, Bristol Myers Squibb, Eli Lilly, Janssen, Merck Sharpe & Dohme, Novartis, Pfizer, and Roche; and has received unconditioned research support from AstraZeneca, Roche, and Merck Sharpe & Dohme. XC, MS, PL, and AF are employed by AstraZeneca. SR has received institutional funding for research support from Amgen, AstraZeneca, Bristol Myers Squibb, Merck, and Pfizer.

The authors declare that this study was funded by AstraZeneca. In July 2020, Daiichi Sankyo entered into a global development and commercialization collaboration with AstraZeneca for datopotamab deruxtecan (Dato-DXd). The funder had the following involvement in the study: study design, collection, analysis, interpretation of data, writing of this article and decision to submit it for publication.

The reviewer LB declared a past co-authorship with the author GP to the handling editor.”

The correct **Conflict of interest** statement is “TN-D has participated in advisory boards with AbbVie, Amgen, AstraZeneca, Bayer, Bristol Myers Squibb, Boehringer Ingelheim, Daiichi Sankyo, EQRx, Gilead, GlaxoSmithKline, Janssen, Eli Lilly, Merck, Merck Sharpe & Dohme, Novartis, Novocure, Pfizer, Regeneron, Roche, Sanofi, and Takeda; has been an invited speaker for Amgen, AstraZeneca, Bristol Myers Squibb, Boehringer Ingelheim, EQRx, Gilead, GlaxoSmithKline, Janssen, Eli Lilly, Novartis, Roche, and Takeda; and has been a steering committee member for AstraZeneca, Merck Sharpe & Dohme, and Roche. BM has participated in advisory boards with Amgen, AstraZeneca, Boehringer Ingelheim, Bristol Myers Squibb, GlaxoSmithKline, Janssen, Merck, Novartis, Prizer, Regeneron, Roche, and Sanofi. RH has participated in advisory boards with Amgen, AstraZeneca, Daiichi Sankyo, Biohaven, Claim, Eli Lilly, Novartis, Regeneron, Sanofi, Merck, and Gilead; and has received research funding institutional from AstraZeneca, Daiichi Sankyo, Eli Lilly, Mirati, Novartis, Corvus, and Mythic. SL has acted in an advisory role for AstraZeneca, Pfizer, Hutchison MediPharma, ZaiLab, GenomiCare, Novartis, Yuhan Corporation, Menarini, Mirati Therapeutics Inc, Daiichi Sankyo, Inc., D3 Bio Limited, Simcere, Takeda and Roche; has been an invited speaker for AstraZeneca, Roche, and Hansoh; is a member of the board of directors for Innovent Biologics, Inc.; has received research grants from AstraZeneca, Hutchison, Bristol Myers Squibb, Heng Rui, BeiGene, Roche, and Hansoh; and has participated in a Speaker’s bureau for AstraZeneca, Roche, and Hansoh. GP has participated in advisory boards for, received honoraria and speaker’s fees from, and acted as a consultant for Amgen, AstraZeneca, Bristol Myers Squibb, Eli Lilly, Janssen, Merck Sharpe & Dohme, Novartis, Pfizer, and Roche; and has received unconditioned research support from AstraZeneca, Roche, and Merck Sharpe & Dohme. XC, MS, PL, and AF are employed by AstraZeneca. SR has received institutional funding for research support from Amgen, AstraZeneca, Bristol Myers Squibb, Merck, and Pfizer.

The authors declare that this study was funded by AstraZeneca. The funder had the following involvement in the study: study design, collection, analysis, interpretation of data, writing of this article and decision to submit it for publication.

The reviewer LB declared a past co-authorship with the author GP to the handling editor.”

An incorrect **Funding** statement was provided. The correct funding statement reads:

“The author(s) declared that financial support was received for this work and/or its publication. The TROPION-Lung10 study (NCT06357533) is sponsored by AstraZeneca. In July 2020, Daiichi Sankyo entered into a global development and commercialization collaboration with AstraZeneca for datopotamab deruxtecan (Dato-DXd).”

The original version of this article has been updated.

